# Prediction of unpowered diving and floating for large-depth manned submersible based on geometric similarity

**DOI:** 10.1038/s41598-026-56191-4

**Published:** 2026-06-04

**Authors:** Zhonghui Hu, Shuai Liu, Wenxin Qu, Lei Jiang, Cong Ye

**Affiliations:** 1 China Ship Scientific Research Center, Wuxi, 214082 China; 2 State Key Laboratory of Deep-sea Manned Vehicles, Wuxi, 214082 China; 3 Taihu Laboratory of Deepsea Technological Science, Wuxi, 214082 China

**Keywords:** Manned submersible, Geometric similarity, Unpowered, Diving, Floating, Prediction, Engineering, Mathematics and computing, Ocean sciences

## Abstract

Large-depth manned submersibles generally conduct diving and floating operations in an unpowered mode. On the premise of guaranteeing satisfactory diving and floating speed, this mode can significantly reduce energy consumption and prolong underwater operating time, which serves as one of the key foundations for the underwater operational performance of manned submersibles. Therefore, the study on prediction methods for unpowered diving and floating motion is of great engineering significance. At present, existing prediction methods are mainly established on the standard motion model, whose results are extremely sensitive to the handling of hydrodynamic terms in the model, thus limiting the prediction accuracy. In this paper, a prediction method for unpowered diving and floating motion of manned submersibles is proposed based on geometric similarity theory, by deriving the relationship between the submersible’s diving and floating speed and its net weight in water. Taking the “Jiao Long” manned submersible as a research object, the established model is employed to predict the 7000 m unpowered diving and floating motion using the 5000 m deep-sea trial data. Comparative analysis indicates that the proposed method achieves higher prediction accuracy than the conventional standard motion model, which preliminarily verifies its feasibility and effectiveness and its general applicability remains to be further examined in follow-up investigations. Meanwhile, many assumptions are adopted in the development of the proposed method. Therefore, this method is only applicable to the quasi-steady segments of the same manned submersible, and is not suitable for transient phases such as water entry, ballast release transitions, or motions involving significant attitude coupling. Furthermore, the method is only intended for operational application phases, rather than the demonstration and design phases of manned submersibles.

## Introduction

Manned submersibles are specialized deep-sea submersible systems capable of transporting scientific and engineering personnel, together with various specialized equipment, to conduct rapid, accurate and sustainable round-trip voyages to multiple seabed locations for high-efficiency detection, sampling and operational missions^1^. It fully leverages the subjective initiative and creativity of on-site professionals, serving as one of the critical pieces of equipment for marine access, exploration, development, and conservation^2^. During the 1980 s, multiple manned submersibles, such as the American “Alvin”^3^, the Russian “MIR-1” and “MIR-2”^4,5^, the Japanese “Shinkai 6500”^6^, and the French “Nautile”^7^, were commissioned. With operating depths mainly concentrated between 2,000 m and 6,500 m, they exerted a significant influence on deep-sea applications such as scientific research, environmental investigation, resource prospecting, and salvage operations. The American “Alvin” conducted detailed investigations on the seabed in the Galápagos Rift region, confirmed the existence of deep-sea hydrothermal vents. The Russian Mir submersible carried Russian and American scientists and jointly discovered the largest known hydrothermal ore deposit to date in the Atlantic Ocean, which was named after the Mir submersible. Japanese scientists aboard the “Shinkai 6500” identified faults on the slope of the Japan Trench at a depth of 6200 m and carried out comprehensive geological surveys. France and other partners implemented the large international “KM3NeT” project, in which the “Nautile” was used to maintain the detector array of the neutrino telescope undersea observatory, advancing frontier interdisciplinary research such as particle astrophysics. Since the 21 st century, the development of manned submersibles, especially full-ocean-depth manned submersibles with an operating depth of 11,000 m, has sparked a new round of scientific and technological competition. In 2012, the manned submersible “Deepsea Challenger”, developed by an American team led by James Cameron, descended to a depth of 10,898 m in the Mariana Trench, establishing a new depth record for solo dives^8^. On November 10, 2020, China’s indigenously developed full-ocean-depth manned submersible “Fen Dou Zhe” successfully descended to a depth of 10,909 m in the Mariana Trench, establishing a new record for China’s manned deep-sea diving operations^9^.

Diving and floating are essential stages in the operation of large-depth manned submersibles, with unpowered diving and floating motion as the primary method. Diving and floating ballast consists of load counterweights arranged on both sides of the submersible and serves as a critical component for enabling the manned submersible to perform unpowered diving and floating motion at a predetermined speed. Specifically, the diving ballast is employed to achieve the unpowered diving motion of the manned submersible, and the floating ballast to realize its unpowered floating motion. The detailed procedure is as follows. Prior to the commencement of operational missions, unpowered diving and floating motion prediction shall be performed to verify whether the weight of the onboard diving and floating ballast is sufficient to enable the manned submersible to achieve the intended diving and floating speed. On the deck of the mother ship, the validated ballast is installed at the mounting interface of the ballast release device. Deploy the manned submersible into the sea. The manned submersible began its dive from the water surface, entering the unpowered diving phase. Upon reaching the target depth, the operator manipulates the ballast release device via the control system to jettison the ballast, bringing the manned submersible into a force-balanced state. When the manned submersible has completed underwater operations, the operator uses the control system to actuate the ballast release device and jettison the ballast, allowing the submersible to enter the unpowered floating phase. Under energy-constrained conditions, this locomotion mode can significantly reduce the energy consumption of the manned submersible, reserving more energy for effective operations and ensuring sufficient operational endurance^10–12^. Prediction of unpowered diving and floating motion constitutes an essential approach for realizing the accurate configuration of diving and floating ballast, and directly affects the control of motion velocity. Regarding the investigation of prediction methodologies, Liu Zhengyuan et al. developed a mathematical model for diving and floating motion of submersibles grounded on the standard motion model, thereby realizing the prediction of unpowered diving and floating maneuvers for manned submersibles^13^. Building on the foundation laid in literature^13^, Pan Binbin et al. introduced the concept of variable volume shrinkage ratio to further refine the kinematic mathematical model, and developed an unpowered diving and floating motion analysis system for manned submersibles^14^. All the aforementioned methods are formulated on the basis of the standard motion model. The hydrodynamic terms of the motion model are predominantly characterized by polynomial expressions or derived from the results of hydrodynamic scaled model tests. Due to the large angle of attack and strong nonlinearity inherent in the unpowered diving and floating motion of manned submersibles, the conventional polynomial expressions fall outside the valid range of polynomial expansion. This leads to increased computational errors in the hydrodynamic terms, resulting in significant deviations in the prediction of the submersible’s unpowered diving and floating motion. If the hydrodynamic terms are represented based on hydrodynamic model test results, the scale effects between the full-scale submersible and the scaled model will impose stringent requirements on the parameter configuration of model tests and the processing of experimental data.

To reduce the adverse impact of hydrodynamic term processing strategies in the standard motion model on the prediction accuracy of unpowered diving and floating motion, this study establishes a novel prediction method for the unpowered diving and floating motion of manned submersibles. Based on the force analysis of manned submersibles, the geometric similarity theory is introduced to derive the quantitative relationship between diving and floating velocity and the underwater gravity of the submersible. In this paper, the “Jiao Long” manned submersible is taken as the research object. Based on the 5000 m sea trial data, a prediction model for the unpowered diving and floating motion of the “Jiao Long” manned submersible is obtained through training. Subsequently, the established unpowered diving and floating motion prediction method is used to predict the 7000 m unpowered diving and floating motion of the “Jiao Long” manned submersible. The feasibility and effectiveness of the unpowered diving and floating motion prediction method based on geometric similarity theory are validated through the comparison of prediction results. This provides a possible technical means for the accurate prediction of unpowered diving and floating motions in practical applications.

## Prediction modeling of unpowered diving and floating motion based on geometric similarity

### Hypothesis

The research work is carried out in the context of engineering applications. The hypothesis for simplified calculation is given in literature^15^, as follows:

1. During the unpowered diving and floating motion of the submersible, only the variation of the velocity in the vertical direction is considered.
2. The unpowered diving and floating motion of a manned submersible is approximated as constant velocity motion, whereby the acceleration of the unpowered diving and floating motion is approximated to be zero.
3. In the calculation process, only the smooth dive phase and the smooth float phase of the manned submersible are considered.

### Force analysis

The authors have comprehensively analyzed the forces acting on the manned submersible during unpowered diving and floating motion, together with the corresponding calculation methods, in literature^16^. For brevity, these details are not repeated here, and the results are presented directly as follows.

**Fig. 1:**
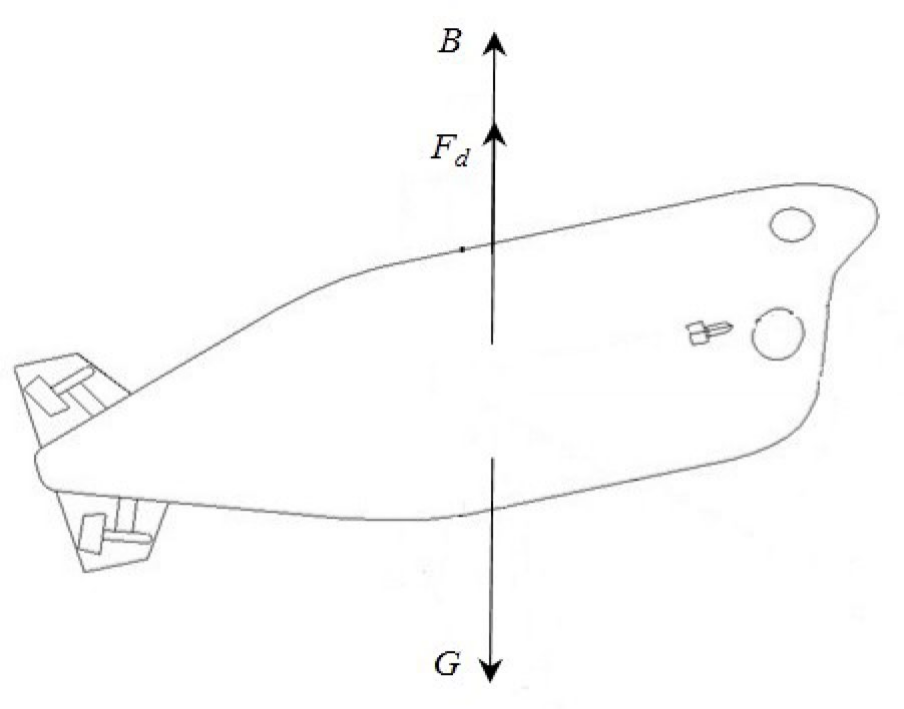
Force analysis.

The forces acting on the manned submersible during unpowered diving and floating motion are illustrated in Fig. 1.

As illustrated in Fig. 1, the forces exerted on the manned submersible during unpowered diving and floating motions comprise gravity, buoyancy, and hydrodynamic forces. The relationships among these forces are as follows:

1 $$\vec{G} + \vec{B} + \vec{F}_{d} = M\vec{a}$$

In the equation, *M* denotes the weight of the manned submersible, and *a* represents its acceleration during unpowered diving and floating motion.

In light of the assumptions in Sect. 2.1, Eq. (1) can be further simplified as:

2 $$\left\{ \begin{gathered} \Delta \vec{G} + \vec{F}_{d} = 0 \hfill \\ \Delta \vec{G} = \vec{G} + \vec{B} \hfill \\ \end{gathered} \right.$$

In the equation, Δ*G* represents the underwater gravity of the manned submersible.

#### Gravity *G*

The weight *M* of the manned submersible is composed of the fixed weight of the submersible, the weight of pilots, the weight of consumables inside the manned tank, the weight of tools, the weight of diving and floating ballast, and the weight of other temporarily loaded equipment and payloads. The gravitational force *G* on the manned submersible is expressed as:

3 $$G = \left( {M_{0} + M_{p} + M_{c} + M_{t} + M_{d} + M_{f} + M_{{other}} } \right) \cdot g$$

In the equation, *M*_0_ denotes the fixed weight of the submersible, *M*_*p*_ the weight of pilots, *M*_*c*_ the weight of consumables inside the manned tank, *M*_*t*_ the weight of tools, *M*_*d*_ the weight of diving ballast, *M*_*f*_ the weight of floating ballast, and *M*_*other*_ the weight of other temporarily mounted payloads.

#### Buoyancy *B*

During unpowered diving and floating, seawater density and hydrostatic pressure vary with depth. Changes in hydrostatic pressure induce variations in the displacement volume of the manned submersible. Consequently, the buoyancy force *B* acting on the manned submersible is expressed as:

4 $$\left\{ \begin{gathered} B = \rho g\left( {1 - k} \right)V_{0} \hfill \\ k = k_{1} + k_{2} + k_{3} \hfill \\ \end{gathered} \right.$$

In the equation, *ρ* denotes the density of seawater. *g* denotes the gravitational acceleration. *V*_0_ denotes the displaced volume when the manned submersible is completely submerged at the start of the dive at the sea surface. *k* denotes the coefficient of displaced volume loss, which is defined as the ratio of the displaced volume lost by the submersible to *V*_0_. *k*_1_ denotes the displaced volume loss coefficient of the pressure-resistant structure, which is defined as the ratio of the displaced volume lost by the pressure-resistant structure of submersible to *V*_0_. *k*_2_ denotes the displaced volume loss coefficient of the buoyant material, which is defined as the ratio of the displaced volume lost by the buoyant material of submersible to *V*_0_. *k*_3_ denotes the displaced volume loss coefficient of the pressure-compensating component, which is defined as the ratio of the displaced volume lost by the pressure-compensating component of submersible to *V*_0_. As documented in the literature^17^, the density of seawater at depths calculated via the EOS80 seawater equation of state.

##### The coefficient of displaced volume loss for pressure-resistant structures

The displaced volume loss coefficient of the pressure-resistant structure *k*_1_ is expressed as:

5 $$k_{1} = k_{{11}} + k_{{12}}$$

In the equation, *k*_11_ denotes the displaced volume loss coefficient of the spherical pressure-resistant structure, *k*_12_ the displaced volume loss coefficient of the cylindrical pressure-resistant structure.

The displaced volume loss coefficient of the spherical pressure-resistant structure *k*_11_ is expressed as:

6 $$\left\{ \begin{gathered} k_{{11}} = \frac{{\sum {\frac{{4\pi }}{3}\left[ {(r_{{Si}} + t_{{Si}} + \Delta r_{{Si1}} )^{3} - (r_{{Si}} + t_{{Si}} + \Delta r_{{Si0}} )^{3} } \right]} }}{{V_{0} }} \hfill \\ \Delta r_{{Si0}} = \frac{{P_{{Si}} \cdot (r_{{Si}} + t_{{Si}} /2)^{2} }}{{2E_{{Si}} \cdot t_{i} }} \cdot (1 - \mu _{{Si}} ) \hfill \\ \Delta r_{{Si1}} = \frac{{(P_{{Si}} - P) \cdot r_{{Si}}^{2} }}{{2E_{{Si}} \cdot t_{{Si}} }} \cdot (1 - \mu _{{Si}} ) \hfill \\ P = \rho \cdot g \cdot h \hfill \\ \end{gathered} \right.$$

In the equation, *r*_*S*_ denotes the inner radius of the spherical shell, *t*_*S*_ the thickness, Δ*r*_*S*0_ the radial deformation at the onset of diving, Δ*r*_*S*1_ the radial deformation at depth *h*, *E*_*S*_ the Young’s modulus of the spherical shell material, *µ*_*S*_ the Poisson’s ratio of the spherical shell material, *P*_*S*_ the internal pressure acting on the spherical pressure-resistant structure, *P* the seawater pressure at depth *h*, and *h* the operating depth of the manned submersible.

The displaced volume loss coefficient of the cylindrical pressure-resistant structure *k*_12_ is expressed as:

7 $$\left\{ \begin{gathered} {k_{21}}=\frac{{\sum {\pi \left[ {{{({r_{Ci1}}+{t_{Ci}}+\Delta {r_{Ci1}})}^2}({l_{Ci}}+\Delta {l_{Ci1}}) - {{({r_{Ci1}}+{t_{Ci}}+\Delta {r_{Ci0}})}^2}({l_{Ci}}+\Delta {l_{Ci0}})} \right]} }}{{{V_0}}} \hfill \\ \Delta {r_{Ci0}}=\frac{{2 - {\mu _{Ci}}}}{{{E_{Ci}}}} \times \frac{{{P_{Ci}} \cdot r_{{Ci1}}^{2} \cdot {r_{Ci2}}}}{{r_{{Ci2}}^{2} - r_{{Ci1}}^{2}}} \hfill \\ \Delta {l_{Ci0}}=\frac{{1 - 2{\mu _{Ci}}}}{{{E_{Ci}}}} \times \frac{{{P_{Ci}} \cdot r_{{Ci1}}^{2} \cdot {l_{Ci}}}}{{r_{{Ci2}}^{2} - r_{{Ci1}}^{2}}} \hfill \\ \Delta {r_{Ci1}}=\frac{{1 - 2{\mu _{Ci}}}}{{{E_{Ci}}}} \times \frac{{{P_{Ci}} \cdot r_{{Ci1}}^{2} - P \cdot r_{{Ci2}}^{2}}}{{r_{{Ci2}}^{2} - r_{{Ci1}}^{2}}} \cdot {r_{Ci2}}+\frac{{1+{\mu _{Ci}}}}{{{E_{Ci}}}} \times \frac{{{P_{Ci}} - P}}{{r_{{Ci2}}^{2} - r_{{Ci1}}^{2}}}r_{{Ci1}}^{2} \cdot {r_{Ci2}} \hfill \\ \Delta {l_{Ci1}}=\frac{{1 - 2{\mu _{Ci}}}}{{{E_{Ci}}}} \times \frac{{{P_{Ci1}} \cdot r_{{Ci1}}^{2} - P \cdot r_{{Ci2}}^{2}}}{{r_{{Ci2}}^{2} - r_{{Ci1}}^{2}}} \cdot {l_{Ci}} \hfill \\ P=\rho \cdot g \cdot h \hfill \\ \end{gathered} \right.$$

In the equation, *r*_*C*1_ denotes the inner radius of the cylindrical shell, *r*_*C*2_ the outer radius, *l*_*C*_ the axial length, *t*_*C*_ the thickness, Δ*r*_*C*0_ the radial deformation at the onset of diving, Δ*l*_*C*0_ the axial deformation at the onset of diving, Δ*r*_*C*1_ the radial deformation at diving depth *h*, Δ*l*_*C*1_ the axial deformation at depth *h*, *E*_*C*_ the Young’s modulus of the cylindrical shell material, *µ*_*C*_ the Poisson’s ratio of the cylindrical shell material, and *P*_*C*_ the internal pressure of the cylindrical pressure-resistant structure.

##### The coefficient of displaced volume loss for buoyant materials

The displaced volume loss coefficient of the buoyant material *k*_2_ is expressed as:

8 $$k_{2} = k_{{21}} + k_{{22}}$$

In this context, *k*_21_ represents the displaced volume loss coefficient caused by the absorption of water of the buoyant material, whereas *k*_22_ represents the displaced volume loss coefficient caused by the volume contraction of the buoyant material.

The volume loss of buoyant material due to water absorption can be expressed as the coefficient of buoyancy loss, designated *k*_21_, which can be expressed as follows:

9 $$k_{{21}} = \frac{{\rho _{{bm}} \cdot V_{{bm0}} \cdot \eta _{{bm}} }}{{\rho \cdot V_{0} }}$$

In this context, *ρ*_*bm*_ denotes the density of the buoyant material, and *V*_*bm*0_ denotes the initial displaced volume of the buoyant material, *η*_*bm*_ represents the water absorption fraction of the buoyant material.

The volume loss of buoyant material due to volume shrinkage can be expressed as the buoyancy loss coefficient, designated *k*_22_, which can be expressed as follows:

10 $$\left\{ \begin{gathered} k_{{22}} = - \frac{P}{{K_{{bm}} }} \cdot \frac{{V_{{bm0}} }}{{V_{0} }} \hfill \\ P = \rho \cdot g \cdot h \hfill \\ K_{{bm}} = \frac{{E_{{bm}} }}{{3\left( {1 - 2\mu _{{bm}} } \right)}} \hfill \\ \end{gathered} \right.$$

In this context, *K*_*bm*_ represents the volumetric elastic modulus, *E*_*bm*_ denotes the Young’s modulus of the buoyant material, *µ*_*bm*_ the Poisson’s ratio of the buoyant material.

##### The coefficient of displaced volume loss for the pressure-compensating component

The displaced volume loss coefficient of the pressure-compensating component is *k*_3_ expressed as:

11 $${k_3}={\eta _{hf}} \cdot \frac{{{V_{hf0}}}}{{{V_0}}}$$

In this context, *η*_*hf*_ represents the compression fraction of a hydraulic fluid, *V*_*hf*0_ represents the volume of hydraulic fluid at atmospheric pressure.

### Motion prediction model

The hydrodynamic forces acting on a manned submersible can be expressed as follows:

12 $${F_d}=\frac{1}{2}\rho {v^2}S \cdot {C_Z}$$

In this context, *ρ* represents the density of seawater, *S* denotes the wetted surface area of the deep-sea manned submersible, *v* denotes the velocity in the dive and float directions, and *C*_*z*_ denotes the drag coefficients in the diving and floating directions. During the unpowered diving and floating motion of the manned submersible, its velocity changes slightly, resulting in a relatively small variation of the Reynolds number. Accordingly, the drag coefficients in the diving and floating directions *C*_*z*_ is assumed to be constant in this study.

Based on the geometric similarity theory presented in literature^18^, the relationships between the wetted surface area *S*, displaced volume *V*, and characteristic length *l* of the manned submersible are respectively given by:

13 $$S \propto l^{2}$$

14 $$V \propto l^{3}$$

According to Eqs. (13) and (14), the relationship between the wetted surface area *S* and displaced volume *V* of the manned submersible is given by:

15 $$S \propto {l^2} \propto {\left( {{V^{1/3}}} \right)^2} \propto {V^{2/3}}$$

It is assumed that the variation in seawater density and the change in the displacement volume of the manned submersible with depth are small quantities. Meanwhile, for the same manned submersible, the weight variation is approximately equal to the equivalent displaced mass. According to the scaling law in geometric similarity and the above assumptions, the following relation holds:

16 $$\Delta G \propto V \propto {l^3}$$

The relationship between the wetted surface area *S* of the manned submersible and its underwater gravity Δ*G* can be obtained as:

17 $$S \propto {l^2} \propto {\left( {\Delta {G^{1/3}}} \right)^2} \propto \Delta {G^{2/3}}$$

Substituting Eq. (12) into Eq. (2) yields:

18 $$\Delta G=\frac{1}{2}\rho {v^2}S \cdot {C_Z}$$

Substituting Eq. (17) into Eq. (18) yields:

19 $$\Delta G=\frac{1}{2}\rho {v^2}S \cdot {C_Z} \propto \frac{1}{2}\rho {v^2} \cdot {C_Z} \cdot \Delta {G^{2/3}} \Rightarrow {v^2} \propto \frac{2}{{\rho \cdot {C_Z}}} \cdot \Delta {G^{1/3}}$$

Together with the aforementioned assumptions that the change in seawater density *ρ* is a small quantity and the drag coefficients in the diving and floating directions *C*_*z*_ remains constant, we obtain:

20 $${v^2} \propto \Delta {G^{1/3}}$$

Taking the square root of both sides gives:

21 $$v \propto \Delta {G^{1/6}}$$

According to Eq. (21), for the same manned submersible, the diving and floating velocity *v* of the manned submersible is proportional to the 1/6 power of its underwater gravity Δ*G*. Based on this, the prediction model for the diving and floating velocity *v* can be established as:

22 $$\left\{ \begin{gathered} \vec{v} = b \cdot \Delta \vec{G}^{{1/6}} + c \hfill \\ \Delta \vec{G} = \vec{G} + \vec{B} \hfill \\ G = \left( {M_{0} + M_{p} + M_{c} + M_{t} + M_{{ad}} + M_{{other}} } \right) \cdot g \hfill \\ B = \rho \cdot g \cdot \left( {1 - k} \right) \cdot V_{0} \hfill \\ \end{gathered} \right.$$

In this equation, the gravitational force *G* acting on the manned submersible is calculated using the method presented in Sect. 2.2.1. The buoyant force *B* exerted on the manned submersible is determined by the approach described in Sect. 2.2.2. Parameters *b* and *c* are the coefficients of the prediction model, which are identified from the training dataset.

It can be seen from Eq. (22) that:

1. Parameter *b* can be interpreted as the velocity gain coefficient, which reflects the sensitivity of the velocity in the diving and floating directions to the net underwater gravity and represents the response intensity of diving and floating speed to net gravity. It is jointly determined by the drag coefficients in the diving and floating directions *C*_*z*_, seawater density *ρ*, and wetted surface area *S*. A larger *b* indicates a more significant influence of net gravity on the diving and floating velocity.
2. Parameter *c* can be regarded as the velocity correction term. Since the proposed method is based on the smooth phase of diving and floating motion, an initial velocity value exists accordingly.

## Parameter identification method for motion prediction model

In this paper, the least squares method is adopted to identify the parameters b and c in Eq. (22). Let:

23 $$\left\{ \begin{gathered} x = \Delta \vec{G}^{{1/6}} \hfill \\ y = \vec{v} \hfill \\ \end{gathered} \right.$$

Substituting Eq. (23) into Eq. (22) yields:

24 *y = b · x + c*

Let the number of training samples be *n*, then for the given *n* samples $\left\{ {\left( {{x_1},{y_1}} \right),\left( {{x_2},{y_2}} \right),...,\left( {{x_i},{y_i}} \right)} \right\}$,*i=1,2, … ,n*, the sum of squared errors *R* can be expressed as:

25 $$R = \sum\limits_{{i = 1}}^{n} {\left( {y_{i} - b \cdot x_{i} - c} \right)^{2} }$$

Based on the least squares criterion, it follows that:

26 $$\left\{ \begin{gathered} \sum\limits_{{i = 1}}^{n} {\left( {y_{i} - b \cdot x_{i} - c} \right)x_{i} } = 0 \hfill \\ \sum\limits_{{i = 1}}^{n} {\left( {y_{i} - b \cdot x_{i} - c} \right)} = 0 \hfill \\ \end{gathered} \right.$$

Equation (24) yields:

27 $$\left\{ \begin{gathered} b = \frac{{n\sum {x_{i} y_{i} } - \sum {x_{i} } \sum {y_{i} } }}{{n\sum {x_{i}^{2} - \left( {\sum {x_{i} } } \right)^{2} } }} \hfill \\ c = \frac{{\sum {x_{i}^{2} \sum {y_{i} } } - \sum {x_{i} y_{i} } \sum {x_{i} } }}{{n\sum {x_{i}^{2} - \left( {\sum {x_{i} } } \right)^{2} } }} \hfill \\ \end{gathered} \right.$$

## Prediction method for unpowered diving and floating motion

**Fig. 2:**
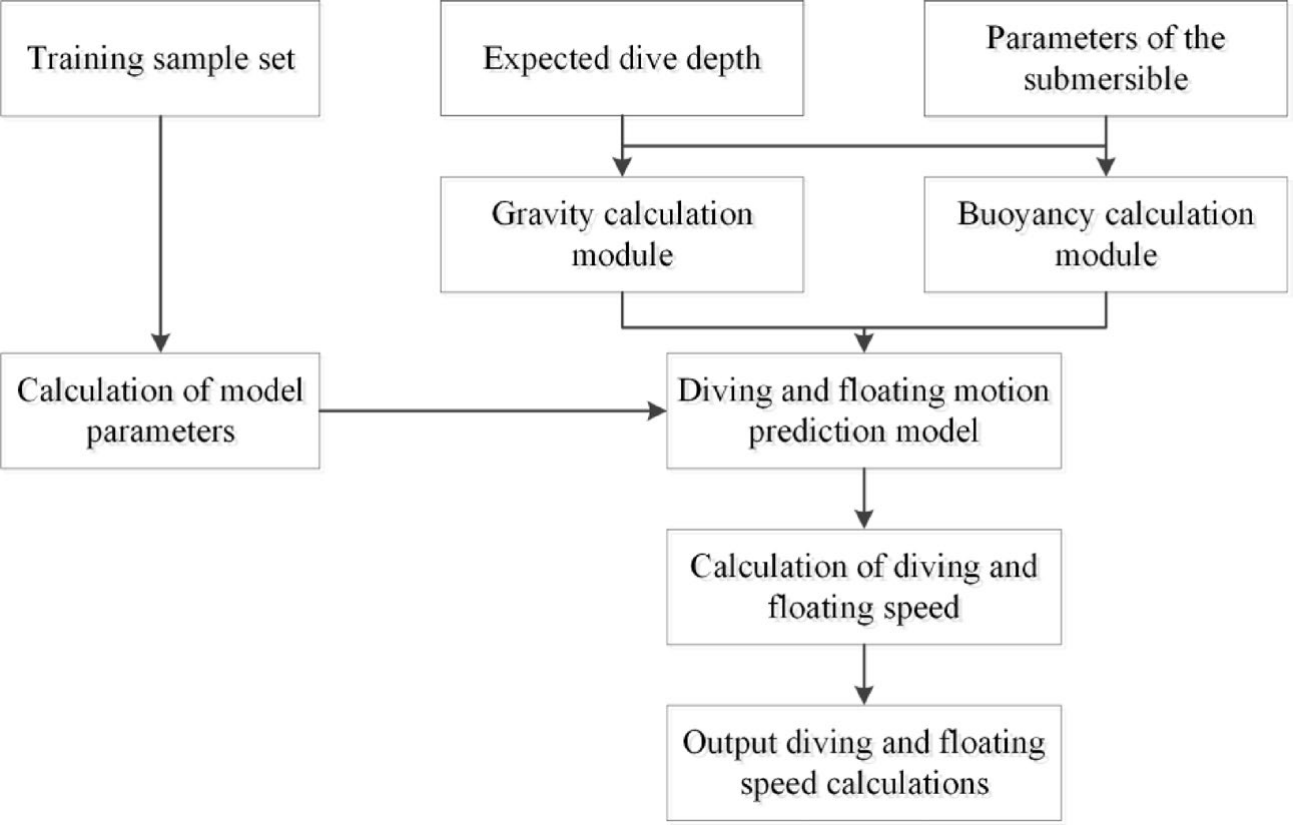
Overall calculation flow.

The prediction method for the unpowered diving and floating motion of the manned submersible is illustrated in Fig. 2. First, the parameters *b* and *c* in the prediction model are identified using Eq. (27) based on the training dataset constructed from historical data, thereby establishing the unpowered diving and floating motion prediction model. Then, the diving and floating motion is calculated according to the parameters of the manned submersible and the desired diving/floating velocity. The calculation includes the diving phase and the floating phase, both of which use the same algorithm. The calculation procedure is illustrated below taking the diving phase as an example. The calculation is performed iteratively with depth as the iterative variable. At the start of the calculation, the initial depth is set to zero, which is implemented by the depth increment module. Based on the submersible parameters and the current depth, the gravitational force is computed by the gravity calculation module, and the buoyant force is obtained by the buoyancy calculation module. The calculated gravitational and buoyant forces are then input into the established unpowered diving and floating motion prediction model to obtain the velocity at the current depth. Afterwards, the depth is increased by a predefined step, and the above procedure is repeated to obtain velocities at different depths until the target depth is reached.

**Fig. 3:**
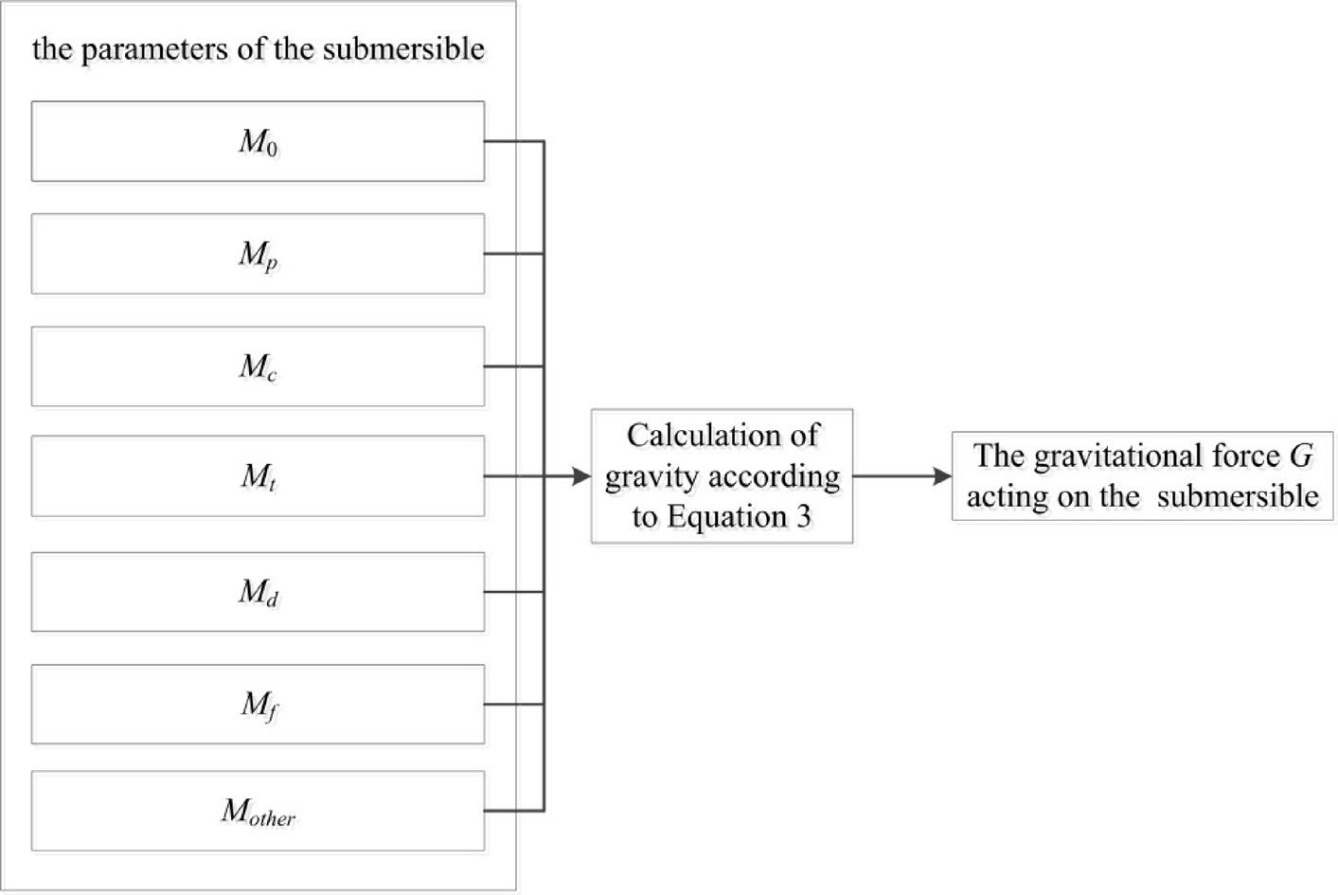
Gravity calculation module.

The methodology for the gravity calculation module in Fig. 2 is shown in Fig. 3. Using the weight parameters of the manned submersible, the gravitational force is evaluated via Eq. (3).

Key parameters for this calculation are summarized in Table 1.

**Table 1:** The parameters necessary to calculate the gravity force.

Parameters	Parameter usage
The fixed weight of the submersible *M*_0_	These parameters are utilised for the purpose of calculating the variable gravity *G* of the submersible.
The weight of pilots *M*_*p*_	
The weight of consumables in the manned tank *M*_*c*_	
The weight of tools *M*_*t*_	
The weight of the diving and floating ballast *M*_*d*_	
The weight of the diving and floating ballast *M*_*f*_	
Other weights *M*_*other*_	

**Fig. 4:**
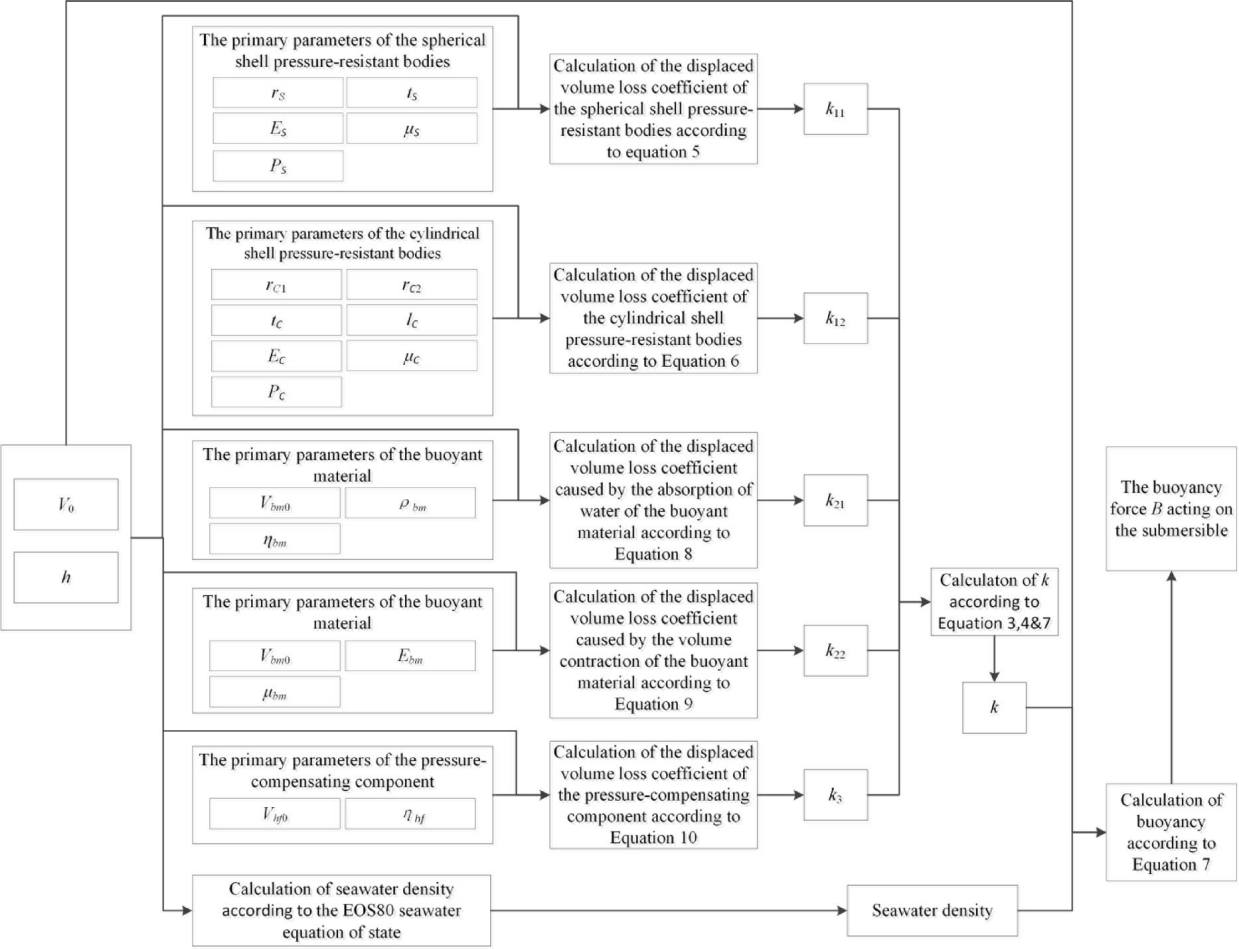
Buoyancy calculation module.

The buoyancy calculation module in Fig. 2 is detailed in Fig. 4. Specifically, the volume loss coefficients are first derived using Eqs. (4)–(11), based on the properties of the spherical and cylindrical pressure-resistant structures, buoyancy material, and hydraulic oil. Subsequently, the seawater density at the current operating depth is calculated using the EOS80 equation of state. The buoyant force is then determined according to Eq. (4).

Parameters required for buoyancy estimation are presented in Table 2.

**Table 2:** The parameters necessary to calculate the buoyancy force.

Parameters		Parameter usage
The displaced volume when the manned submersible is completely submerged at the start of the dive at the sea surface *V*_0_		This parameter is utilised for the purpose of calculating the displaced volume loss coefficient *k*.
The current depth of the submersible *h*		This parameter is utilised for the purpose of calculating process parameters such as seawater pressure and seawater density, derived from the current depth.
The primary parameters of the spherical shell pressure-resistant bodies	Radius *r*_*S*_	These parameters are utilised for the purpose of calculating the displaced volume loss coefficient *k*_11_ of the spherical shell pressure-resistant bodies.
	Wall thickness *t*_*S*_	
	Young’s modulus *E*_*S*_	
	Poisson’s ratio *µ*_*S*_	
The primary parameters of the cylindrical shell pressure-resistant bodies	Inner radius *r*_*C*1_	These parameters are utilised for the purpose of calculating the displaced volume loss coefficient *k*_12_ of the cylindrical shell pressure-resistant bodies.
	Outer radius *r*_*C*2_	
	Wall thickness *t*_*C*_	
	Length *l*_*C*_	
	Young’s modulus *E*_*C*_	
	Poisson’s ratio *µ*_*C*_	
The primary parameters of the buoyant material	Volume at atmospheric pressure *V*_*bm*0_	These parameters are utilised for the purpose of calculating the displaced volume loss coefficient *k*_21_ caused by the absorption of water of the buoyant material and the displaced volume loss coefficient *k*_22_ caused by the volume contraction of the buoyant material.
	Density *ρ*_*bm*_	
	Water absorption fraction *η*_*bm*_	These parameters are utilised for the purpose of calculating the displaced volume loss coefficient *k*_21_ caused by the absorption of water of the buoyant material.
	Young’s modulus *E*_*bm*_	These parameters are utilised for the purpose of calculating the displaced volume loss coefficient *k*_22_ caused by the volume contraction of the buoyant material.
	Poisson’s ratio *µ*_*bm*_	
The primary parameters of the pressure-compensating component	Volume of hydraulic fluid at atmospheric pressure *V*_*hf*0_	These parameters are utilised for the purpose of calculating the displaced volume loss coefficient of the pressure-compensating component *k*_3_.
	Compression fraction of hydraulic fluid *η*_*hf*_	

## Computational validation

In this paper, the “Jiao Long” manned submersible is taken as the research object. The unpowered diving and floating motion is predicted using the prediction model proposed in this study, and the predicted results are compared with the sea trial data and the prediction results from literature^16^, which is based on the standard motion model. Specifically, the 5000 m diving test data of the D44 dive of the “Jiao Long” submersible are utilized as the training samples, whereas the 7000 m diving test data of the D55 dive and the results from literature^16^ are employed as the validation samples.

### Main parameters of the “Jiao Long” manned submersible

The key parameters of the “Jiao Long” manned submersible provided by literature^15^ are summarized in Table 3.

**Table 3:** The primary parameters of the “Jiao Long” manned submersible.

Parameter term	Value
The fixed weight *M*_0_	20,414 kg
The fixed displaced volume *V*_0_	20.8047 m^3^
Displaced volume loss coefficient of the pressure-resistant structure *k*_1_	3.65 × 10^− 7^*h*
Displaced volume loss coefficient of the buoyant material *k*_2_	2.23 × 10^− 6^*h*
Displaced volume loss coefficient of the pressure-compensating component *k*_3_	2.67 × 10^− 7^*h*

### Identification of prediction model parameters

**Fig. 5:**
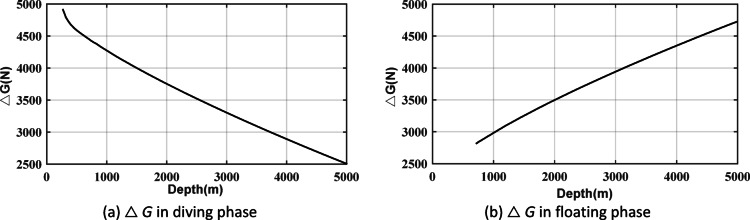
△*G* curve of “Jiao Long” with depth during 5000 m diving test.

In this paper, the parameters of the prediction model are identified based on the 5000 m diving test data of the D44 dive of the “Jiao Long” manned submersible presented in literature^16^. During the 5000 m diving test of the D44 dive, the variation of the in-water gravity of the “Jiao Long” submersible with depth during the unpowered diving and floating motion is illustrated in Fig. 5.

The parameters in Eq. (22) are identified using Eq. (27), and the identification results are listed in Table 4.

**Table 4:** Prediction model parameter identification results.

Motion phase	Parameter term	Value
Diving	*b*	0.3171
	*c*	−0.6210
Floating	*b*	0.3842
	*c*	−0.9442

Substituting the identified parameters into Eq. (22) yields the unpowered diving and floating motion prediction model of the “Jiao Long” manned submersible as follows:

28 $$v = \left\{ \begin{gathered} 0.3171 \cdot \left( {G - B} \right)^{{1/6}} - 0.621,diving \hfill \\ 0.3842 \cdot \left( {G - B} \right)^{{1/6}} - 0.9442,floating \hfill \\ \end{gathered} \right.$$

### 7000 m unpowered diving and floating motion prediction

The 7000 m unpowered diving and floating motion of the “Jiao Long” manned submersible is predicted using Eq. (27). Payload conditions for the submersible during the dive 50 are given in Table 5.

**Table 5:** The payload parameters of dive 50.

Payload term		Weights (kg)	Displaced volume (m^3^)
Consumables in the manned tank		35	0
Pilots		224	0
Tools		35	0.0588
Diving ballast		246	0.0315
Floating ballast		600	0.0769
Other	Ballast water	176	0
	Lead blocks for equalization	203	0.0174

**Fig. 6:**
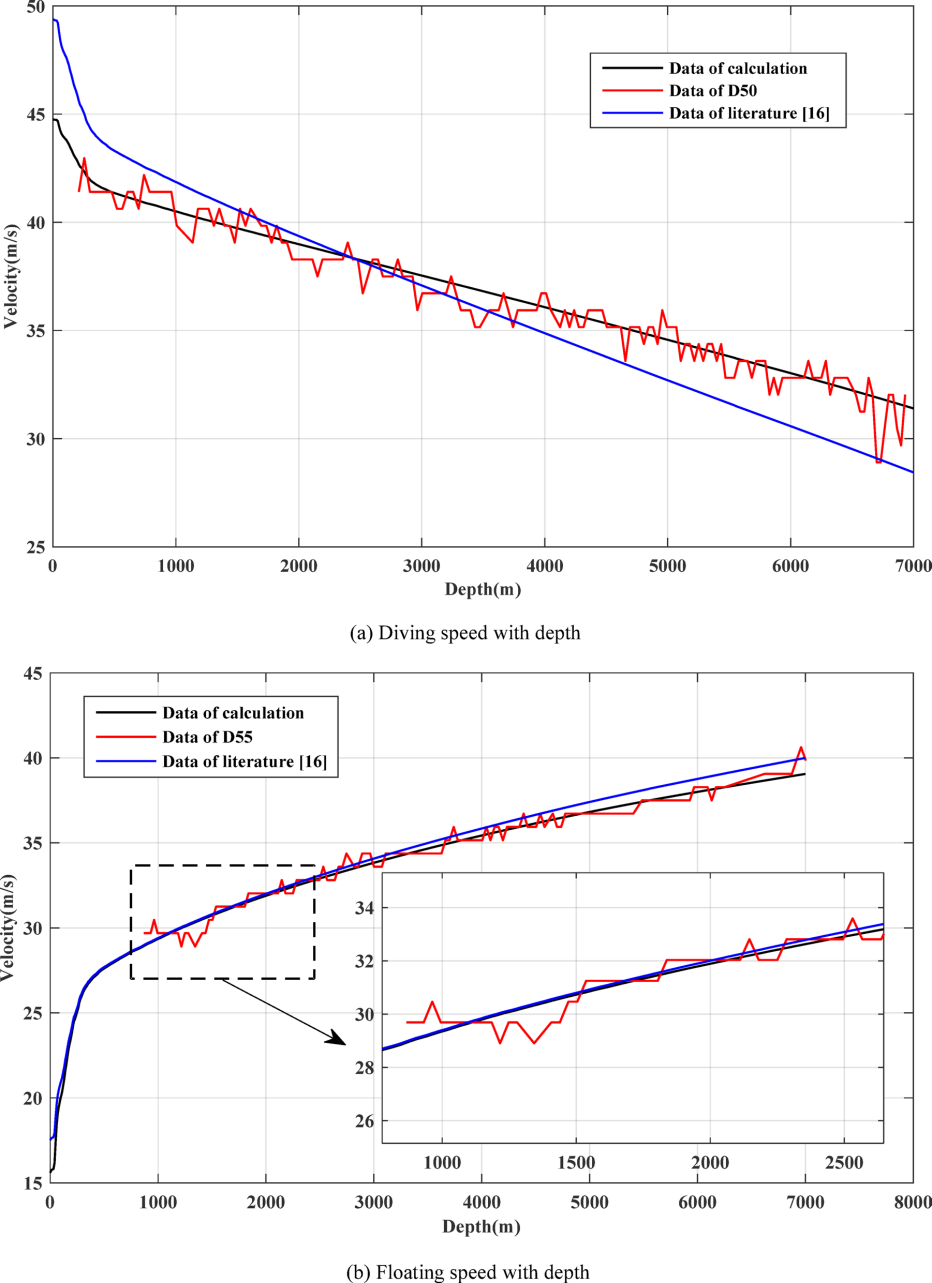
Predicted velocity curves of 7000 m unpowered diving and floating motion.

The corresponding prediction results are illustrated in Fig. 6.

It can be observed from Fig. 6 that the predicted results are highly consistent with the sea trial data in terms of variation trend. Compared with the prediction results in literature^16^, the proposed method yields better agreement with the experimental measurements than the standard motion model.

### Error comparison and analysis

**Fig. 7:**
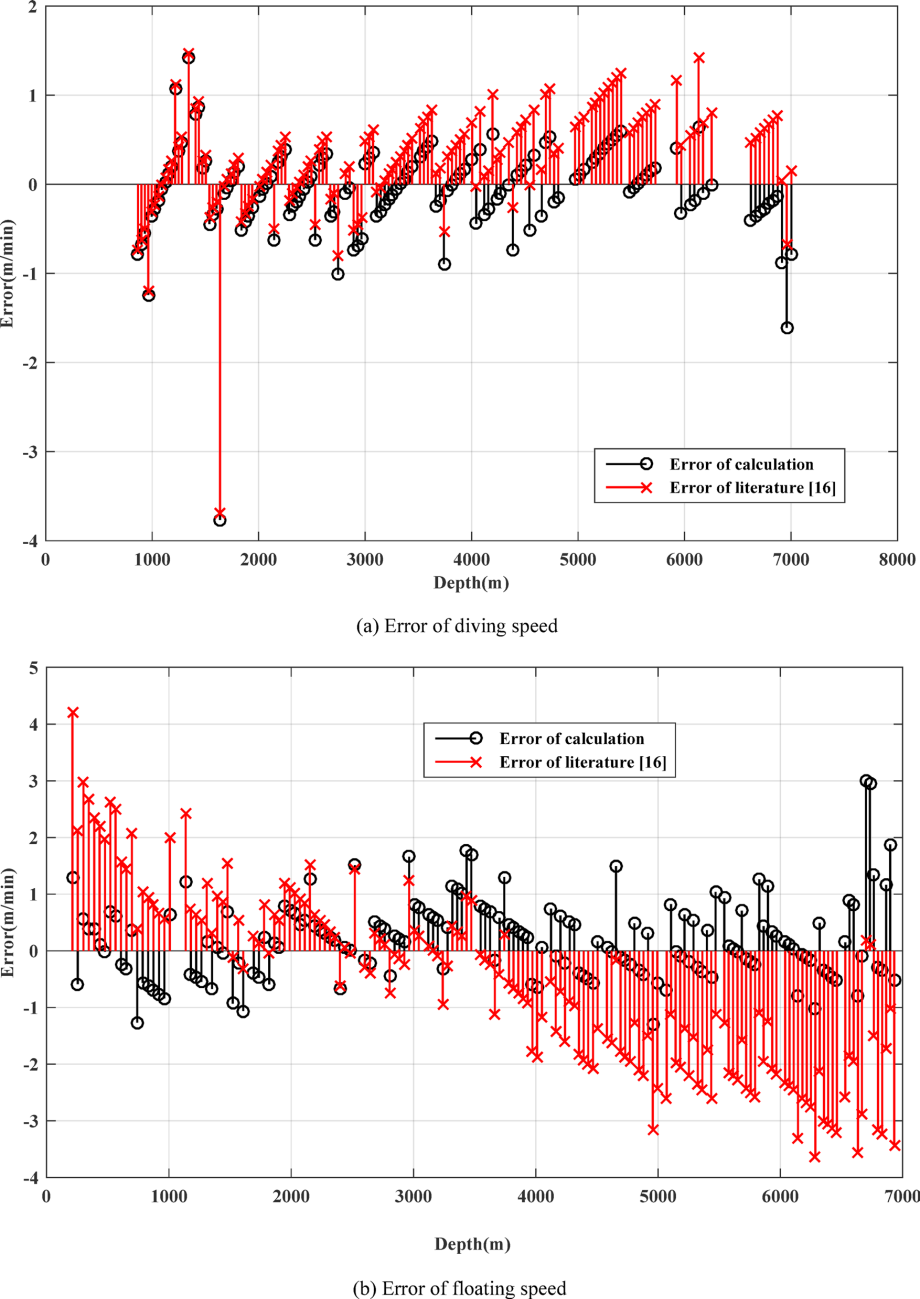
Comparison of error in prediction of diving and floating speed.

The error comparison of the 7000 m unpowered diving and floating motion prediction is displayed in Fig. 7.

The error comparison of the prediction results is summarized in Table 6. The error metrics adopted include root mean square error (RMSE), maximum absolute error, mean absolute error (MAE), maximum relative error, and average relative error.

**Table 6:** Comparison of error of prediction between two models.

Types of error		Prediction method based on geometric similarity	Prediction method based on standard dynamic model
RMSE	Diving speed	0.73 m/min	1.71 m/min
	Floating speed	0.41 m/min	0.58 m/min
Maximum absolute error	Diving speed	2.99 m/min	4.21 m/min
	Floating speed	1.41 m/min	1.47 m/min
MAE	Diving speed	0.56 m/min	1.41 m/min
	Floating speed	0.32 m/min	0.48 m/min
Maximum relative error	Diving speed	10.36%	10.88%
	Floating speed	4.89%	5.09%
Average relative error	Diving speed	1.58%	3.98%
	Floating speed	0.94%	1.38%

In accordance with the error comparison results presented in Fig. 7; Table 6, a comparative error analysis is carried out between the prediction method based on geometric similarity and that based on standard dynamic model.

As illustrated in Fig. 7, during the diving phase, the prediction error associated with the standard dynamic model rises monotonically with increasing depth, whereas that of the geometric similarity–based method remains considerably steadier with noticeably smaller fluctuations. In the floating phase, the prediction errors of both methods display mild, stable variations with a narrow performance gap, and the geometric similarity–based method still yields a marginal yet consistent advantage.

As revealed in Table 6, the RMSE, MAE, and average relative error of the geometric similarity–based prediction method are all lower than those of the standard dynamic model–based method, indicating that its prediction results are generally closer to the measured data. In terms of root-mean-square error, the reduction reaches 57.3% for diving speed and 29.3% for floating speed, demonstrating that the geometric similarity–based method can significantly suppress large errors and achieve superior overall stability. With respect to maximum absolute error and maximum relative error, the maximum absolute error for diving speed is reduced by 28.9%, while that for floating speed remains nearly equivalent with a slight advantage; the maximum relative errors of the two methods are close and both controlled at a low level, implying better control over extreme deviations and higher stability for the proposed method. Regarding mean absolute error and average relative error, the mean absolute error is reduced by 60.2% for diving speed and 33.3% for floating speed; the average relative error is reduced by 60.3% for diving speed and 31.8% for floating speed, confirming that the geometric similarity–based method provides higher overall accuracy and better agreement with actual conditions.

The errors of the geometric similarity–based prediction method mainly arise from:

1. The method only considers quasi-steady segments and ignores transient phases such as initial water entry, ballast release transitions, and motions involving significant attitude coupling.
2. When introducing the geometric similarity theory, it is assumed that the variation in seawater density and the change in the displacement volume of the manned submersible with depth are small quantities, and the drag coefficient is approximated as a constant.

## Conclusion

Based on the force analysis of manned submersibles, this paper introduces the geometric similarity theory to establish a prediction model and develop a corresponding method for the unpowered diving and floating motion of manned submersibles. Taking the “Jiao Long” manned submersible as the research object, the parameters in the prediction model are identified using the least squares method based on 5,000 m sea trial data and the unpowered diving and floating motion at 7000 m is predicted, which preliminarily validates the feasibility and effectiveness of the proposed method. Nevertheless, its general applicability remains to be further examined in follow-up investigations. Based on the aforementioned validation results, a comprehensive comparison is conducted between the proposed method and existing representative approaches from four perspectives: model assumptions, hydrodynamic dependence, model complexity, and applicable scope. The detailed comparison results are presented in Table 7.

**Table 7:** Comparison of prediction methods for unpowered diving and floating motion.

Method	Assumptions	Hydrodynamic dependence	Model complexity	Applicable scope
Literature^13^	6-DOF motion polynomial fitting	High (sensitive to hydrodynamic term processing)	High	Full motion phase (including transient)
Literature^15^	Vertical motion Quasi-steady segments	Medium (depends on model test data)	Medium	The smooth dive and float phase
Proposed method	Vertical motion Quasi-steady segments	Low (weakened by geometric scaling)	Low	The smooth dive and float phase

It can be seen from Table 7 that after introducing the geometric similarity theory:

1. The influence of hydrodynamic forces in the prediction model is weakened, and the dependence of prediction accuracy on the processing methods of hydrodynamic terms is reduced.
2. The complexity of the model is lowered, making the prediction more concise and convenient.
3. The applicable scope of the method is limited. The method is only suitable for quasi-steady segments and not for transient phases such as initial entry, ballast release transitions, or motions involving significant attitude coupling.

Meanwhile, many assumptions are adopted in the development of the proposed method, and its validation is only preliminary due to the limitation of test samples. Therefore, the method still has the following limitations:

1. The prediction method for unpowered diving and floating motion of manned submersibles based on geometric similarity theory is only suitable for the application phase, rather than the demonstration and design phases of manned submersibles.
2. In the prediction model, the transferability of parameters is limited. Parameters can only be transferred to the same manned submersible but cannot be directly transferred to manned submersibles with different shapes and main dimensions, for which re-identification is required.
3. Although the method demonstrates higher prediction accuracy in the case study, its general applicability remains to be further examined in follow-up investigations.

## Data Availability

The datasets generated and analyzed during the current study are not publicly available due to workplace and laboratory regulations and confidentiality requirements. The processed relevant data are available from the corresponding author upon reasonable request.
